# Changes of physiological characteristics, element accumulation and hormone metabolism of tea leaves in response to soil pH

**DOI:** 10.3389/fpls.2023.1266026

**Published:** 2023-11-16

**Authors:** Xiaoli Jia, Qi Zhang, Yuhua Wang, Ying Zhang, Mingzhe Li, Pengyuan Cheng, Meihui Chen, Shaoxiong Lin, Jishuang Zou, Jianghua Ye, Haibin Wang

**Affiliations:** ^1^College of Tea and Food, Wuyi University, Wuyishan, China; ^2^College of Life Sciences, Fujian Agriculture and Forestry University, Fuzhou, China; ^3^College of Life Science, Longyan University, Longyan, China

**Keywords:** tea tree, soil pH, physiological characteristics, multi-element, hormone metabolome

## Abstract

Soil acidification is very likely to affect the growth of tea trees and reduce tea yield. In this study, we analyzed the effects of soils with different pH on the physiological characteristics of tea leaves and determined the multi-element content and hormone metabolomes of tea leaves by ICP-MS and LC-MS/MS, based on which we further analyzed their interaction. The results showed that increasing soil pH (3.29~5.32) was beneficial to increase the available nutrient content of the rhizosphere soil of tea tree, improve the antioxidant enzyme activity and photosynthesis capacity of tea tree leaves, and promote the growth of tea tree. Orthogonal partial least squares discriminant analysis (OPLS-DA) and bubble characteristics analysis were used to screen key elements and hormones for the effect of pH on tea leaves, which were further analyzed by redundancy analysis (RDA) and interaction network. The results showed that an increase in soil pH (3.29~5.32) favored the accumulation of seven key elements (C, K, Ca, Mg, Mn, P, S) in tea tree leaves, which in turn promoted the synthesis of six key hormones (salicylic acid, salicylic acid 2-O-β-glucoside, tryptamine, 2-oxindole-3-acetic acid, indole-3-acetic acid, trans-zeatin-O-glucoside). It can be seen that the increase in soil pH (3.29~5.32) enhanced the resistance of the tea tree itself, improved the photosynthesis ability of the tea tree, and effectively promoted the growth of the tea tree.

## 1 Introduction

Tieguanyin (*Camellia sinensis*) tea tree is a perennial evergreen plant native to Anxi County, Fujian Province, China (latitude 24°50'-25°26'N, longitude 117°36'-118°17'E). Tea tree is an acidophilic plant and is suitable for cultivation when soil pH is 4.0 - 5.5, unsuitable when soil pH is less than 4.0, and optimal for tea tree cultivation when soil pH is 5.0 - 5.5 (Mohammad et al., 2014; Mehra and Baker, 2017). Lin et al. (2023) analyzed soil pH and its effect on tea yield and quality in 145 tea plantations in Nanjing County, Fujian Province, and found that 82.1% of tea plantations had soil pH < 4.5 and soil pH was significantly and positively correlated with tea yield and quality. Wang et al. (2018) and Wang et al. (2022) found that 37.67% of tea plantation soils in Anxi County, Fujian Province, China, had been acidified (pH < 4.5), and that tea yield and quality tended to decrease after soil acidification. It is evident that soil acidification can significantly reduce the yield and quality of tea and limit the development of the tea industry.

In the early stage of this research team, from the perspective of tea tree rhizosphere soil, we analyzed the effect of soil pH on the growth of the tea tree and found that soil acidification would lead to an increase in soil pathogenic bacteria and a decrease in probiotic bacteria in the rhizosphere soil of the tea tree, a decrease in the nutrient cycling capacity of the soil, and a decrease in the nutrient uptake and utilization capacity of the tea tree, which would lead to a decrease in the yield and quality of the tea leaves (Lin et al., 2022; Ye et al., 2023b). Acidification is an abiotic stress on the plant itself, and under stress conditions, plant uptake of soil elements was altered, for different types of abiotic stress, there were significant differences in the mechanisms of element uptake and utilization by plants (Dhaliwal et al., 2022). For example, under drought stress, *Rheum tataricum* increased the translocation and accumulation of Li, Se, Si, and Mo, which in turn improved the *Rheum tataricum* own antioxidant defense (Golubkina et al., 2022). Heavy metal stress reduced the uptake of P, Mn, Ca, S, K and Mg, antioxidant enzyme activities and photosynthetic capacity of *Lemna minor* (Alp et al., 2023). Secondly, changes in the ability of plants to absorb and accumulate soil nutrients could directly affect the synthesis of phytohormones (Ampong et al., 2022).

Phytohormones are mainly simple small-molecule organic compounds, they have complex physiological effects, and have important regulatory roles in plant growth and development, while adversity stress affects the synthesis and accumulation of phytohormones, which in turn regulates plant growth (Waadt et al., 2022). Liu et al. (2022) found that increased adversity stress led to higher levels of abscisic acid and jasmonic acid in mulberry, reduced plant resistance, and lowered yield and quality. Shikha et al. (2023) found that under nutrient stress, plants modulated nutrient uptake by regulating the synthesis of jasmonate hormones. Ahmad et al. (2022) found that under salt stress, cotton mainly took increased levels of gibberellin and salicylic acid to improve their resistance to salt stress to secure their yield. It can be seen that significant changes in plant hormones occurred under adversity stress, and there were significant differences in the regulation of plant hormones by different stress modalities (Mukherjee et al., 2022).

Acidification is an adversity stress. Numerous scholars have conducted numerous studies on the effects of acidification on rhizosphere soil microorganisms, tea tree growth and tea quality, and concluded that acidification alters the community structure of soil microorganisms, which, in turn, reduces tea yield and quality (Lin et al., 2022; Jia et al., 2023; Wang et al., 2023). The growth of tea tree is closely related to its ability to absorb nutrients from the soil, which affects the synthesis of hormones and thus the physiological mechanisms of the tea tree. However, little research has been reported on this aspect. In-depth study and revelation of the changes of key hormones and key elements and their interactions in tea tree leaves induced by acidification are of great significance for exogenous regulation of tea tree growth. Accordingly, this study took tea trees planted in soils with different pH values as the research object to analyze the effects of soils with different pH values on the resistance physiological indexes and photosynthetic physiological indexes of tea tree leaves; at the same time, tea tree leaves were collected for leaf multi-element determination and hormone metabolome determination. On this basis, the key elements and hormones significantly affected by soil pH were screened and analyzed for their interactions, with a view to providing a theoretical basis for the exogenous regulation of tea tree growth.

## 2 Materials and methods

### 2.1 Test tea plantation and sample collection

Anxi County, Fujian Province, China, is the origin of Tieguanyin tea tree. Based on our previous study (Wang et al., 2018; Wang et al., 2022; Ye et al., 2023b), Tieguanyin tea plantations located in Longjuan town (latitude 24° 97′ N, longitude 117°93′ E), Anxi County, Quanzhou City, Fujian Province, China was selected as experimental site. The total area of the experimental tea plantation was about 14.5 hm² and contained soils with pH 3.29 (P1, severely acidified soil), 4.74 (P2, suitable soil for planting), and 5.32 (P3, optimal soil for planting).The experimental site has an average altitude of 600 m, an average annual rainfall of 1800 mm, an average annual relative humidity of 80%, and an average annual temperature of 18 °C. In June 2022, photosynthetic physiological indexes of tea trees were measured, with five replicates for each treatment. The specific sampling method was briefly described as follows: three tea trees were randomly selected to determine photosynthetic physiological indexes of functional leaves, and the average value was taken as one replication, and five replications were set for each treatment. Meanwhile, one bud and three leaves and rhizosphere soil of Tieguanyin tea tree planted in soils with different pH were collected, and tea tree leaves were immediately stored in liquid nitrogen while rhizosphere soil was stored in an ice box, and three independent replicates were set up for each sample. Specific sampling method of tea tree leaves was briefly described as follows: 5 tea trees were randomly selected using “S” sampling, one bud and three leaves of the tea tree were collected, and one replicate was set up after sufficient mixing, and three replicates were set up for each treatment. The collected tea tree leaves were used to determine physiological indexes of leaf resistance, multi-element content and hormone metabolome. The sampling method of rhizosphere soil of tea tree is “S” sampling method, i.e., 5 tea trees were randomly selected, litter was removed from the surface layer of soil, soil was dug out layer by layer to a depth of about 40 cm, tea trees were dug out, the adhered soil was shaken off the tea trees, and the soil that was still adhered to the roots was collected, and it was mixed sufficiently, i.e., it was a replication (Zhang et al., 2023a). Three replications were set up for each treatment. The collected tea tree rhizosphere soil was used to determine basic soil physicochemical indexes and elements.

### 2.2 Determination of basic physicochemical indexes of soil

In this study, total nitrogen (TN), total phosphorus (TP), total potassium (TK), available nitrogen (AN), available phosphorus (AP), available potassium (AK) as basic physicochemical indexes were determined, and specific methods were referred to the technical manual “Soil agrochemical analysis methods” (Lu, 2000). Briefly, the collected soil samples were naturally dried and then passed through a 2 mm nylon mesh sieve. TN content was determined by the Kjeldahl method, i.e., the collected soil was detected with concentrated sulfuric acid, and when the decoction cooled down, it was filtered and could be measured directly by Kjeldahl meter. TP content was determined by alkaline dissolution molybdenum antimony colorimetric method, collected soil was mixed with NaOH and subjected to high temperature treatment, then distilled water was used to dissolve the mixture, this mixture was filtered, and the filtrate was added with molybdenum antimony colorimetry and the absorbance was measured at 700 nm and then converted to TP content. TK content was determined by flame photometer method, the collected soil was mixed with NaOH and subjected to high temperature treatment, then distilled water was used to dissolve the mixture, this mixture was filtered and the filtrate was measured directly by flame photometer to obtain TK content. AN content was determined using the alkaline dissolution diffusion method, where the collected soil was leached using 1 mol/L NaOH solution and the extract was titrated using hydrochloric acid and then converted to AN content. AP content was determined by the NaHCO_3_ leaching-molybdenum antimony colorimetric method. The collected soil was leached with 0.5 mol/L NaHCO_3_, leachate was filtered, and molybdenum antimony colorimetric agent was added to leachate, and absorbance was measured at 880 nm, and then converted to AP content. AK content was determined by the ammonium acetate leaching-flame photometric method, the collected soil was leached with 1 mol/L neutral ammonium acetate, and leachate was filtered and then determined directly by flame photometer.

### 2.3 Determination of photosynthetic physiological indexes

The LI-6400XT Portable Photosynthesis System (Li-Cor, Lincoln, NE, USA) was used to determine leaf photosynthetic rate, stomatal conductance, intercellular CO_2_ concentration, and transpiration rate (Zhang et al., 2023b). Photosynthetic indexes were measured from 9:30 a.m. to 11:30 a.m. on a sunny day, with a photon flux density of 1500 μmol/m^2^·s, a spatial environment with a CO_2_ concentration of 370 ppm, leaf temperatures at 26 ~ 27°C, and a vapor pressure deficit (VPD) of less than 1 kPa in the container. The chlorophyll content of leaves was determined using a chlorophyll analyzer (SPAD-502 PLUS, Tokyo, Japan). Each treatment was performed in five independent replicates.

### 2.4 Determination of physiological indexes of resistance

Physiological indicators of resistance in tea leaves were determined by the method of “Principles and Techniques of Plant Physiological Biochemical Experiments” (Wang, 2006), and superoxide dismutase, peroxidase, catalase activity, soluble sugar content, and malondialdehyde content were measured. Briefly, 0.5 g of fresh tea tree leaves were taken and added to 5 mL of pre-cooled 50 mmol/L phosphate extraction buffer (pH 7.0, containing 1% polyvinylpyrrolidone), ground on an ice bath, homogenized at 4 °C, and centrifuged at 12,000 rpm/min for 10 min, and the supernatant was extracted for the physiological indexes. Superoxide dismutase activity was determined by nitroblue tetrazonium chloride ammonium method with an absorbance of 560 nm. Peroxidase activity was determined by guaiacol colorimetric assay at 470 nm. Catalase activity was determined by potassium permanganate titration and enzyme activity was calculated as the amount of decomposed catalase per minute. Soluble sugar content was determined by anthrone colorimetry at 630 nm. Malondialdehyde content was determined by the thiobarbituric acid method at 450, 532, and 600 nm and then converted to its content.

### 2.5 Determination and quantitative analysis of multi-element content of leaves

The collected fresh tea leaves were rinsed with deionized water to remove the adhering dust and impurities, and then the tea leaves were dried at 80°C to constant weight, ground, and passed through a 75 μm nylon mesh for sample digestion and elemental determination. Accurately weigh 0.5 g of sample powder in a high-pressure digestion tank, 5 replicates for each sample, add 5 mL of HNO_3_, screw it tightly and put it into the oven at 185°C for 4 h. After digestion, open the tank to drive the acid for 1 h (Zhang et al., 2021). Wash the digestion tank with deionized water and transfer it to a 50 mL volumetric flask, and then determine the elemental content by inductively coupled plasma mass spectrometry (ICP-MS). Measurements were repeated three times for each sample and averaged.

The instrumental parameters of the inductively coupled plasma mass spectrometer (Nexion 2000, PE, New York, USA) were 1350 W of RF power, carrier gas flow rate of 0.94 L/min, auxiliary gas flow rate of 0.40 L/min, helium flow rate of 4.5 mL/min, the temperature of the nebulization chamber of 2°C, sample lifting rate of 0.3 r/s, sampling depth of 7 mm, dwell time 50 ms, the nebulizer was a PFA nebulizer, the sampling cone was a nickel cone, the acquisition mode was peak hopping, and the number of scans was 6 times (Bai et al., 2021).

The digestion of blank and standard solutions was carried out according to the digestion method of the test samples, and a standard curve was established. Sc, Ge, In, Rh, Re and Bi were used as internal standards. The standard tea control sample (GBW10016) was digested using the same procedure as the sample to validate the analytical method, the analysis was performed three times and each value was calculated as the average of the three measurements, the validation results of the standard sample were shown in **Supplementary Table 1**.

### 2.6 Determination of soil elemental content

Based on previous studies, this study further determined C, Ca, K, Mg, Mn, P, S and Al contents in the soil. The collected soil samples were air-dried at room temperature, ground and passed through a 75μm nylon mesh. A soil sample of 0.5 g was taken and digested according to the digestion method for tea tree leaves in materials and methods (2.5 Determination and quantitative analysis of multi-element content of leaves), and the digested solution was used for ICP-MS determination. Three independent replicates were set up for each sample. The conditions for ICP-MS were the same as those for ICP-MS determination of tea tree leaf digested solution in materials and methods (2.5 Determination and quantitative analysis of multi-element content of leaves). Soil element content was compared using the soil standard substance (GBW07403). The standard substance and sample were subjected to the same digestion procedure and determination method. Three independent replicates were performed for each sample. The test results of C, Ca, K, Mg, Mn, P, S and Al in the soil standard substance were shown in **Supplementary Table 2**.

### 2.7 Determination and quantitative analysis of leaf hormone metabolome

The tea tree leaf samples were individually ground to powder for hormone metabolome assays with three replicates for each sample. The specific assay method was as follows: weigh 50 mg of ground sample, add 10 μL of internal standard mixing solution at a concentration of 100 ng/mL, 1 mL of methanol/water/formic acid (15:4:1, v/v/v) extractant, vortex mixing for 10 min, centrifuge for 5 min at 4°C and 12,000 r/min, and then take the supernatant to be concentrated and fixed with 80% methanol aqueous solution to 100 μL, passed through 0.22 μm filter membrane, and used for LC-MS/MS analysis (Floková et al., 2014; Li et al., 2016).

Data acquisition instrumentation systems for chromatography mass spectrometry consisted primarily of Ultra Performance Liquid Chromatography (ExionLC™ AD, AB Sciex, Concord, Canada) and Tandem Mass Spectrometry (QTRAP^®^ 6500+, AB Sciex, Concord, Canada). Liquid phase conditions were (Xiao et al., 2018), chromatographic column: Waters ACQUITY UPLC HSS T3 C18 column (1.8 µm, 100 mm×2.1 mm i.d.); mobile phase: phase A, ultrapure water (containing 0.04% acetic acid); phase B, acetonitrile (containing 0.04% acetic acid); gradient elution program: at 0 min A/B. The gradient elution program: 95:5 (V/V) for A/B at 0 min, 95:5 (V/V) for A/B at 1.0 min, 5:95 (V/V) for A/B at 8.0 min, 5:95 (V/V) for A/B at 9.0 min, 95:5 (V/V) for A/B at 9.1 min, 95:5 (V/V) for A/B at 12.0 min; flow rate 0.35 mL/min; The flow rate was 0.35 mL/min; the column temperature was 40°C; the injection volume was 2μL. The mass spectrometry conditions were (Šimura et al., 2018), Electrospray Ionization (ESI) temperature 550°C, mass spectrometry voltage 5500 V in the positive ion mode, mass spectrometry voltage -4500 V in the negative ion mode, and Curtain Gas (CUR) 35 psi. in Q-Trap 6500+, each ion pair was scanned based on optimized declustering potential (DP) and collision energy (CE).

Standard solutions of different concentrations of 0.01 ng/mL, 0.05 ng/mL, 0.1 ng/mL, 0.5 ng/mL, 1 ng/mL, 5 ng/mL, 10 ng/mL, 50 ng/mL, 100 ng/mL, 200 ng/mL, 500 ng/mL were prepared (of which L-tryptophan and salicylic acid 2-O-β-glucoside were 20 times of the above concentrations, i.e., the range of standardized concentration was 0.2-10000 ng/mL). The LC-MS/MS detection method was the same as described above, and the corresponding quantitative signal peak intensity data of each concentration standard were obtained sequentially; the standard curves of different hormones were plotted with the external standard to internal standard concentration ratio (Concentration Ratio) as the horizontal coordinate and the peak area ratio of the external standard to the internal standard (Area Ratio) as the vertical coordinate (**Supplementary Table 3**), and the integrated peak area ratios of all detected samples were substituting into the linear equations of the standard curves for the calculations, and finally, the hormone content in the actual samples was obtained.

### 2.8 Statistical analysis

Excel 2017 software was used to categorize raw data and calculate mean and variance. Rstudio 3.3 software was used to create box plots, principal component plots, heat plots, volcano plots, bubble characteristic plots and OPLS-DA model analysis, redundancy analysis, and interaction network analysis.

## 3 Results and discussion

### 3.1 Effect of soil pH on physicochemical indexes of rhizosphere soils of tea trees

In this study, we analyzed the effect of soil pH on the physicochemical indexes of tea tree rhizosphere soil and found that (**Table 1**), the differences of tea tree rhizosphere soils with different pH were not significant in TN, TP, and TK content, whereas there was a significant difference in AN, AP, and AK content. It was shown that with the increase of soil pH (3.29 to 5.32), AN, AP and AK content in the rhizosphere soil of tea tree increased from 35.23 to 91.38 mg/kg, from 3.13 to 15.12 mg/kg and from 49.25 to 127.95 mg/kg, respectively. It has been reported that there is a significant correlation between soil pH and nutrient availability in the rhizosphere soil of tea trees, and that an appropriate increase in soil pH is conducive to promoting nutrient transformation in the rhizosphere soil of tea trees, increasing the content of available nutrients and thus enhancing root activity of tea trees, and promoting tea tree growth (Jia et al., 2023; Ye et al., 2023c). It can be seen that increasing soil pH can effectively promote soil nutrient transformation and increase the available nutrient content of rhizosphere soil of tea trees.

**Table 1:** Basic physicochemical indexes of rhizosphere soil of tea tree.

	P1	P2	P3
Total nitrogen (TN, g/kg)	2.46 ± 0.12 a	2.53 ± 0.09 a	2.49 ± 0.14 a
Total phosphorus (TP, g/kg)	1.24 ± 0.13 a	1.15 ± 0.08 a	1.13 ± 0.09 a
Total potassium (TK, g/kg)	6.84 ± 0.39 a	7.42 ± 0.43 a	7.38 ± 0.65 a
Available nitrogen (AN, mg/kg)	35.23 ± 1.87 c	84.95 ± 1.74 b	91.38 ± 2.86 a
Available phosphorus (AP, mg/kg)	3.13 ± 0.22 c	10.41 ± 0.46 b	15.12 ± 0.77 a
Available potassium (AK, mg/kg)	49.25 ± 2.13 c	113.24 ± 2.94 b	127.95 ± 2.75 a

P1: Soil pH 3.29; P2: Soil pH 4.74; P3: Soil pH 5.32; Means ± standard error (SE) from three replications for each sample is shown; Different lowercase letters indicate that the difference between different samples reaches the p < 0.05 level.

### 3.2 Effect of soil pH on physiological indexes of tea tree leaves

**Figure 1:**
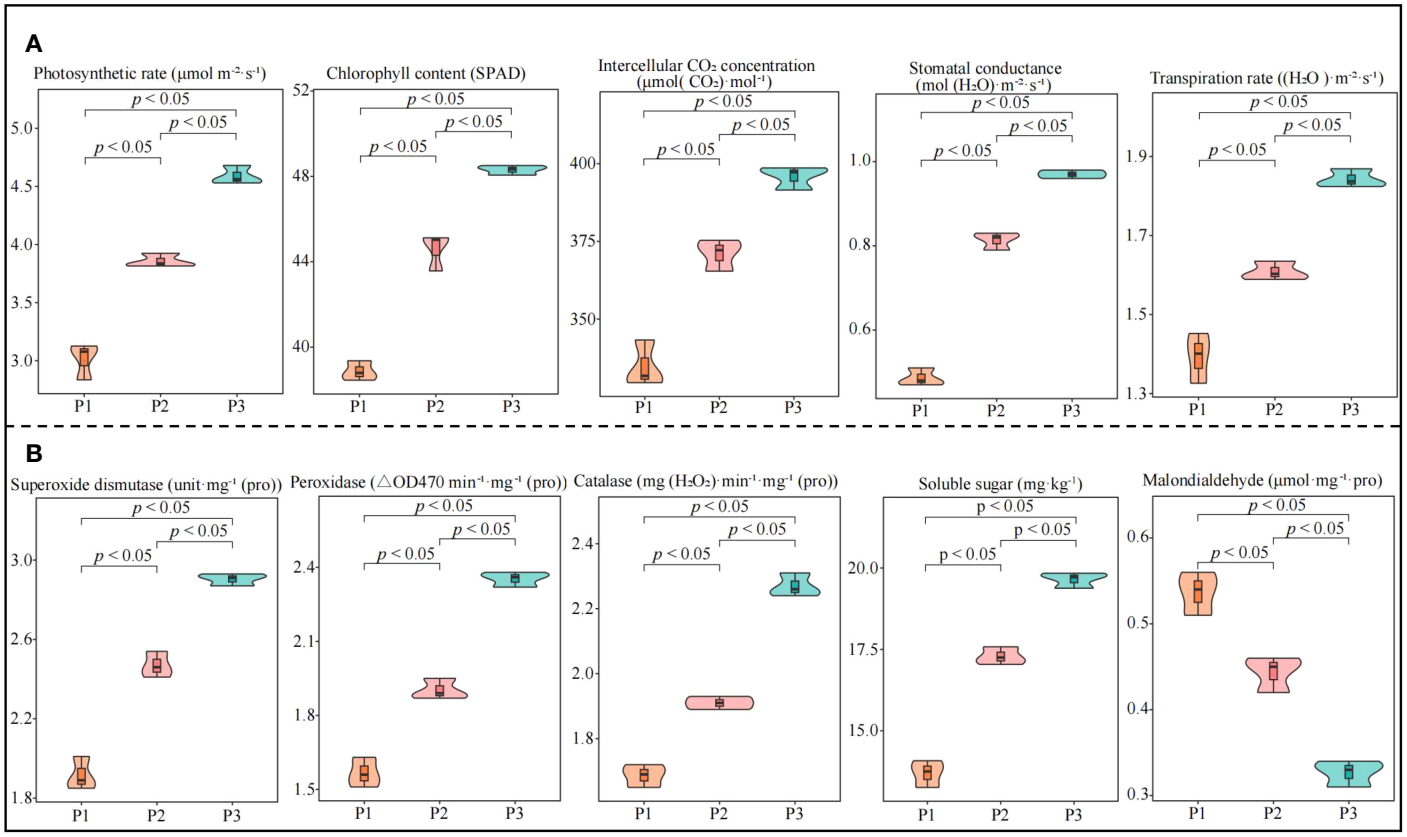
Effect of soil pH on physiological indexes of tea tree leaves. P1: Soil pH 3.29; P2: Soil pH 4.74; P3: Soil pH 5.32; **(A)** Effect of soil pH on photosynthetic physiological indexes of tea leaves; **(B)** Effect of soil pH on resistance physiological indexes of tea leaves.

In this study, we analyzed the effect of soil pH on physiological indexes of tea trees and found that photosynthetic physiological indexes including photosynthetic rate, stomatal conductance, intercellular CO_2_ concentration, transpiration rate, and chlorophyll content of tea leaves showed an increasing trend with increasing soil pH (**Figure 1A**). Among the physiological indexes of resistance in tea leaves, superoxide dismutase, catalase, peroxidase activity and soluble sugar content showed a significant increasing trend with the increase of soil pH, while malondialdehyde content showed a significant decreasing trend (**Figure 1B**). Tea tree is an acidophilic plant, when the soil pH is too low, it is very easy to affect the growth of tea trees, reduce the physiological resistance of tea trees, and then reduce tea yield and quality (Chen et al., 2021; Ye et al., 2022). It has been reported that when soil pH < 4.5, the root growth of the tea tree is inhibited, and the number and area of nascent roots decrease significantly, which in turn contribute to a significant decrease in the biomass of the tea tree (Sun et al., 2020). Damage to the root system of the tea tree directly affects the uptake and utilization of nutrients by the tea tree, which in turn reduces the yield and quality of tea (Ding et al., 2022; Wang et al., 2022). While increasing soil pH is conducive to promoting the growth of tea tree roots, enhancing the root activity and nutrient absorption capacity of tea trees, and improving tea production. It was also found in this study that soil pH significantly affects the physiological characteristics of tea trees, and an appropriate increase in soil pH is beneficial to improve the resistance and photosynthetic capacity of tea trees. It can be seen that the increase in soil pH (3.29-5.32) is conducive to promoting the growth of the root system of the tea tree and improving the nutrient absorption capacity of the tea tree, which in turn improves the photosynthetic capacity and physiological resistance of the tea tree.

### 3.3 Effect of soil pH on multi-element content of tea tree leaves

**Figure 2:**
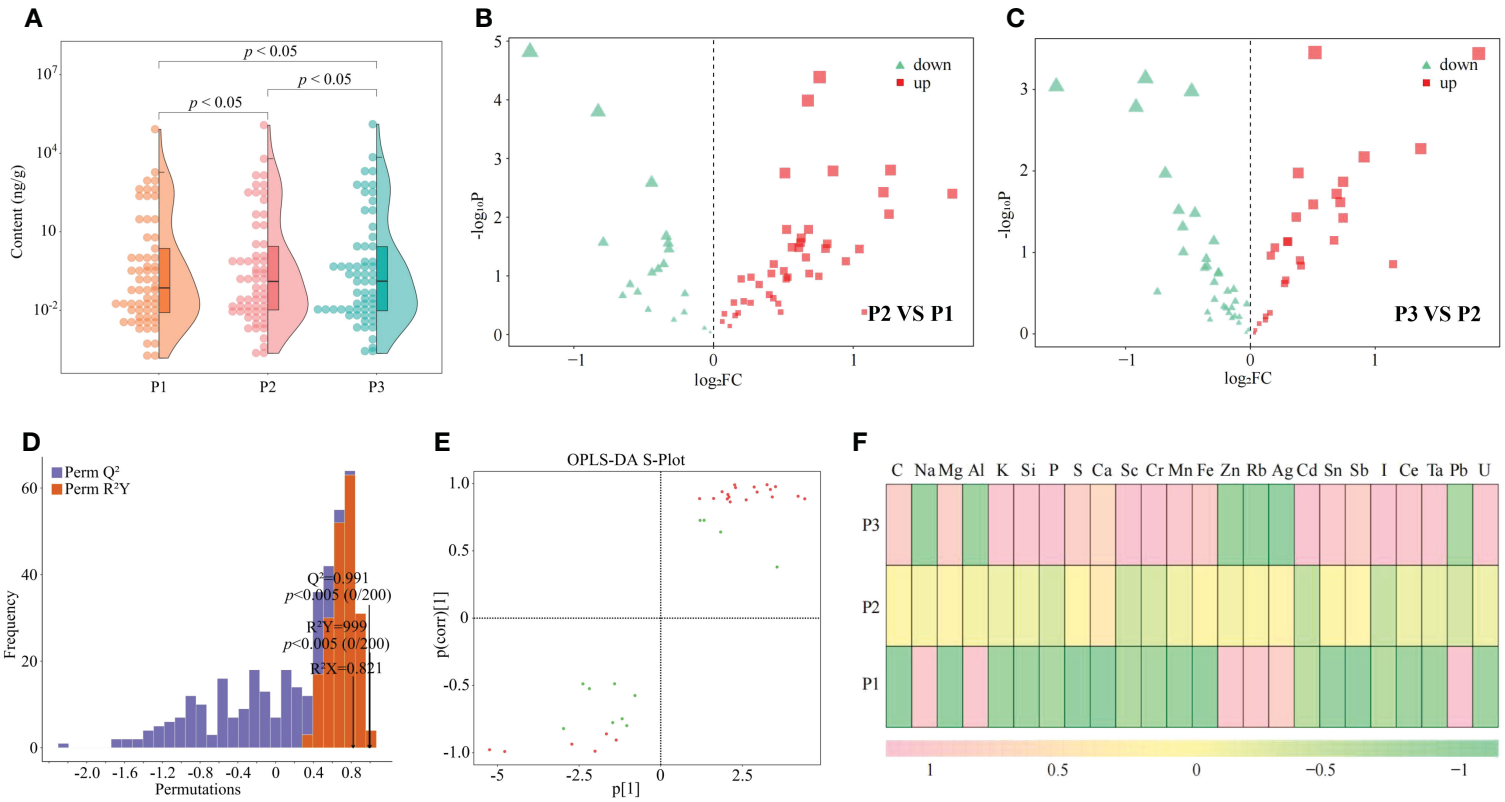
Effect of soil pH on multi-element content of tea tree leaves and screening of key elements. P1: Soil pH 3.29; P2: Soil pH 4.74; P3: Soil pH 5.32; **(A)** Effect of soil pH on the total elemental content of tea tree leaves; **(B)** Volcano plot analysis of the elemental content of tea tree leaves between P2 and P1; **(C)** Volcano plot analysis of the elemental content of tea tree leaves between P3 and P2; **(D)** OPLS-DA model test for screening of key elements in tea samples from soils with different pH; **(E)** S-Plot analysis of element contents of tea samples from soils with different pH based on the OPLS-DA model; **(F)** Analysis of the content of key elements in the leaves of tea trees from soils with different pH.

**Figure 3:**
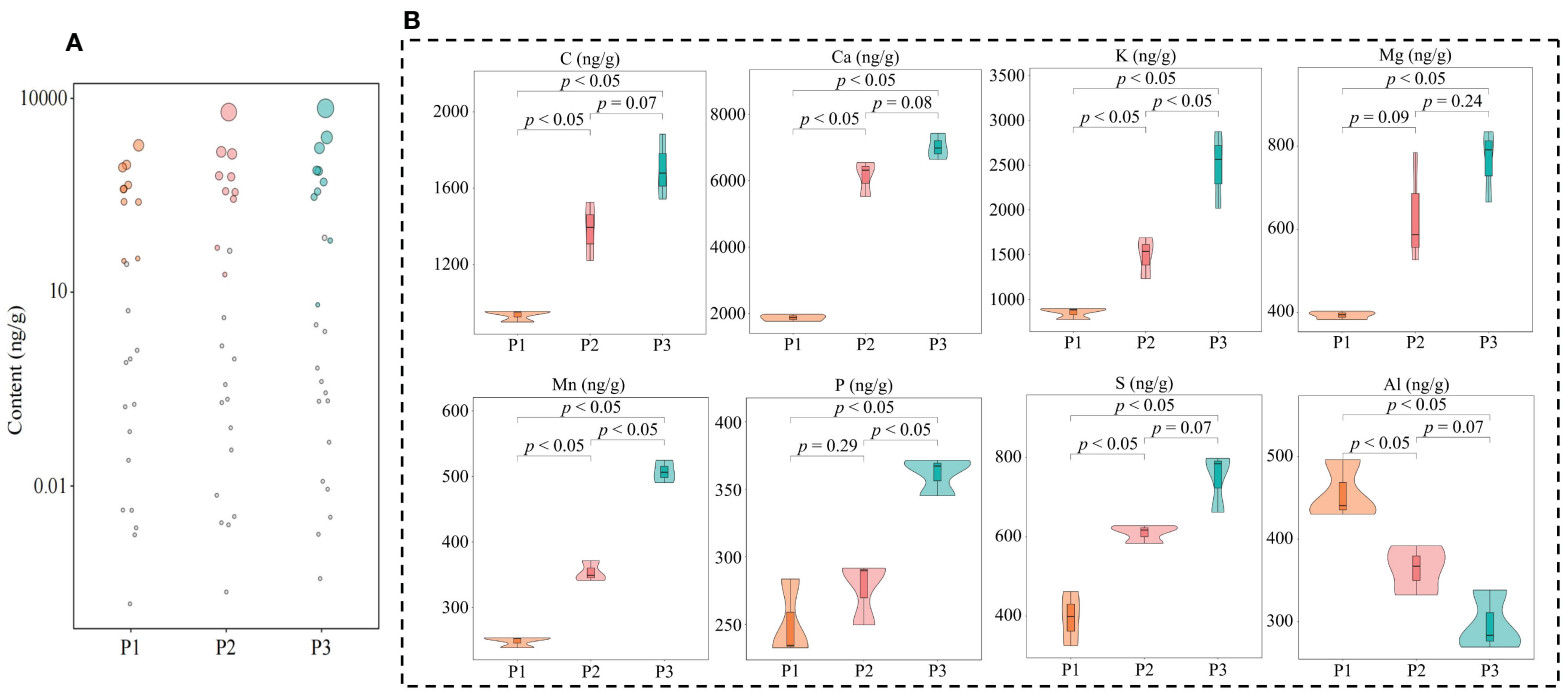
Screening of characteristic elements in tea tree leaves from soils with different pH. P1: Soil pH 3.29; P2: Soil pH 4.74; P3: Soil pH 5.32; **(A)** Screening of characteristic elements of tea tree leaves from soils with different pH based on bubble characteristic plot; **(B)** Content analysis of characteristic elements.

Nutritional elements required for normal growth and development of plants are divided into essential elements and beneficial elements; and essential elements includie massive elements and trace elements, and beneficial elements refer to certain elements required for normal growth and development of plants (Kirkby, 2023). The overall growth and development of plants requires the participation of different elements, which can effectively improve the photosynthesis and respiratory metabolism of plants, increase plant tolerance to various abiotic and biotic stresses, and promote plant growth (Kaur et al., 2023). In this study, it was found that a total of 61 elements were detected in tea tree leaves of soils with different pH, and the total amount of elements in tea tree leaves showed a significant (*p* < 0.05) increasing trend with the increase of soil pH (P1-P3), as shown that at soil pH values of 3.29 (P1), 4.74 (P2), and 5.32 (P3), the elemental content of tea tree leaves was 87.81 mg/g, 129.23 mg/g, and 142.06 mg/g (**Figure 2A**). The results of principal component analysis showed (**Supplementary Figure 1**) that the two principal components effectively distinguished soils with different pH, with a total contribution of 72.3%. It can be seen that soils with different pH can significantly affect the elemental content in tea tree leaves. Accordingly, this study further analyzed the elemental content of tea tree leaves from soils with different pH using volcano diagrams, and the results showed (**Figures 2B, C**) that the content of 22 elements showed a significant increasing trend, while the content of 14 elements showed a significant decreasing trend, with the increase of soil pH. This study further constructed the OPLS-DA model for soils with different pH values based on the elemental contents in tea tree leaves. The results of the goodness-of-fit analysis of the OPLS-DA model showed (**Figure 2D**) that after 200 random simulations, the goodness-of-fit R^2^Y value was 0.999 and the predictability Q^2^ value was 0.991, which were at highly significant levels (*p* < 0.005). It can be seen that the OPLS-DA model constructed in this study has a good fit and high confidence, which can effectively distinguish different samples and can be used for further analysis. The score plot analysis differences between groups of the OPLS-DA model showed (**Supplementary Figure 2**) that the OPLS-DA model could effectively distinguish samples in different regions, and the difference between groups of different samples reached 72.1%. It can be seen that there are significant differences in the elemental content of tea leaves from soils with different pH. The results of S-Plot analysis of OPLS-DA model for soils with different pH showed (**Figure 2E**) that a total of 24 key differential elements were screened and obtained for soils with different pH. The results of content analysis of key elements showed (**Figure 2F**) that the content of 18 elements showed an increasing trend with the increase of soil pH, while the remaining 6 elements showed a decreasing trend. Further bubble characteristic map of 24 key elements in tea tree leaves obtained a total of 8 significantly different characteristic elements (**Figure 3A**), of which the contents of 7 elements (C, Ca, K, Mg, Mn, P, and S) showed a significant upward trend with the increase of soil pH, and the content of 1 element (Al) showed a significant downward trend (**Figure 3B**).

On this basis, this study further determined the contents of eight characteristic elements of tea tree rhizosphere soils at different pH values. The results showed (**Supplementary Table 4**) that the contents of C, Ca, K, Mg, Mn, P, S, and Al were not significantly different among rhizosphere soils with different pH, which varied in the ranges of 135.845~143.257 mg/kg, 2456.275~ 2561.476 mg/kg, 7410.268~7568.327 mg/kg, 89.267~94.365 mg/kg, 24.185~26.184 mg/kg, 128.539~131.287 mg/kg, 286.469~300.432 mg/kg, 358.036~368.186 mg/kg. It can be seen that the differences in the content of the characteristic elements in the leaves of the tea tree are not related to the content of the element in the soil, leading to changes in the content of the key to changes in soil pH caused by changes in the ability of the tea tree to absorb and accumulate the element.

Carbon is an important element for plant growth. It has been reported that an increase in carbon content is conducive to increasing the uptake of elements such as K, Ca, Mg, Mn, P, etc., which is conducive to increasing the photosynthesis capacity and antioxidant capacity of plants (Hu et al., 2022). In this study, it was found that C, Ca, K, Mg, Mn, P, and S in tea tree leaves showed a significant increase with increasing soil pH. It can be seen that increasing soil pH is conducive to increasing C content in tea tree leaves, enhancing plant C metabolism, and promoting the absorption of Ca, K, Mg, Mn, P, and S. K, Ca, Mg, Mn, P and S are all required for plant growth, of which K is an activator of many enzymes in the plant and improves the plant’s ability to photosynthesize and cope with external environmental stresses (Ahammed et al., 2022). Ca is beneficial for improving plant resistance, enhancing the photosynthetic capacity of plants, and promoting plant growth (Huang et al., 2022). Mg and Mn are involved in various physiological and biochemical processes such as photosynthesis, respiratory metabolism, nucleic acid metabolism, etc., and are closely related to crop growth, development and yield (Islam et al., 2022; Kleczkowski and Igamberdiev, 2023). P and S are essential components in the metabolism of nucleic acids and proteins, and are involved in plant respiration and chlorophyll formation, which are important for plant growth (Chtouki et al., 2022; Narayan et al., 2022). It can be seen that with increasing soil pH, the tea tree’s ability to absorb Ca increases, improving the tea tree’s own resistance. The increase in K content is conducive to the activation of a variety of enzyme activities in the tea tree, which in turn promotes the metabolism of the tea tree. The increase of P and S content promotes the synthesis of nucleic acids, proteins, and chlorophyll in the leaves of the tea tree, which in turn promotes the growth and development of the tea tree. However, the increase in Mg and Mn content promotes the photosynthetic capacity of tea trees.

In addition, this study also revealed that Al content showed a significant decreasing trend in tea tree leaves with soil pH increases. Al is not essential for plant growth, and accumulation of Al can damage plant DNA, inhibit root growth, affect plant nutrient uptake, and reduce plant respiration and photosynthesis, thus preventing normal plant growth (Hajiboland et al., 2022). Increasing soil pH reduces the concentration of Al^3+^ in the soil and decreases the uptake and enrichment of Al by plants, while a decrease in the concentration of Al^3+^ also contributes to alleviating soil P fixation and an increasing the uptake of P by plants (Penn and Camberato, 2019). It can be seen that the increase of soil pH is beneficial to increase the accumulation of C, K, Ca, Mg, Mn, P and S in the leaves of tea trees, and reduce the content of Al, which in turn improves the resistance and photosynthesis ability of tea trees and promotes the growth of tea trees.

### 3.4 Effect of soil pH on the hormone metabolome of tea tree leaves

**Figure 4:**
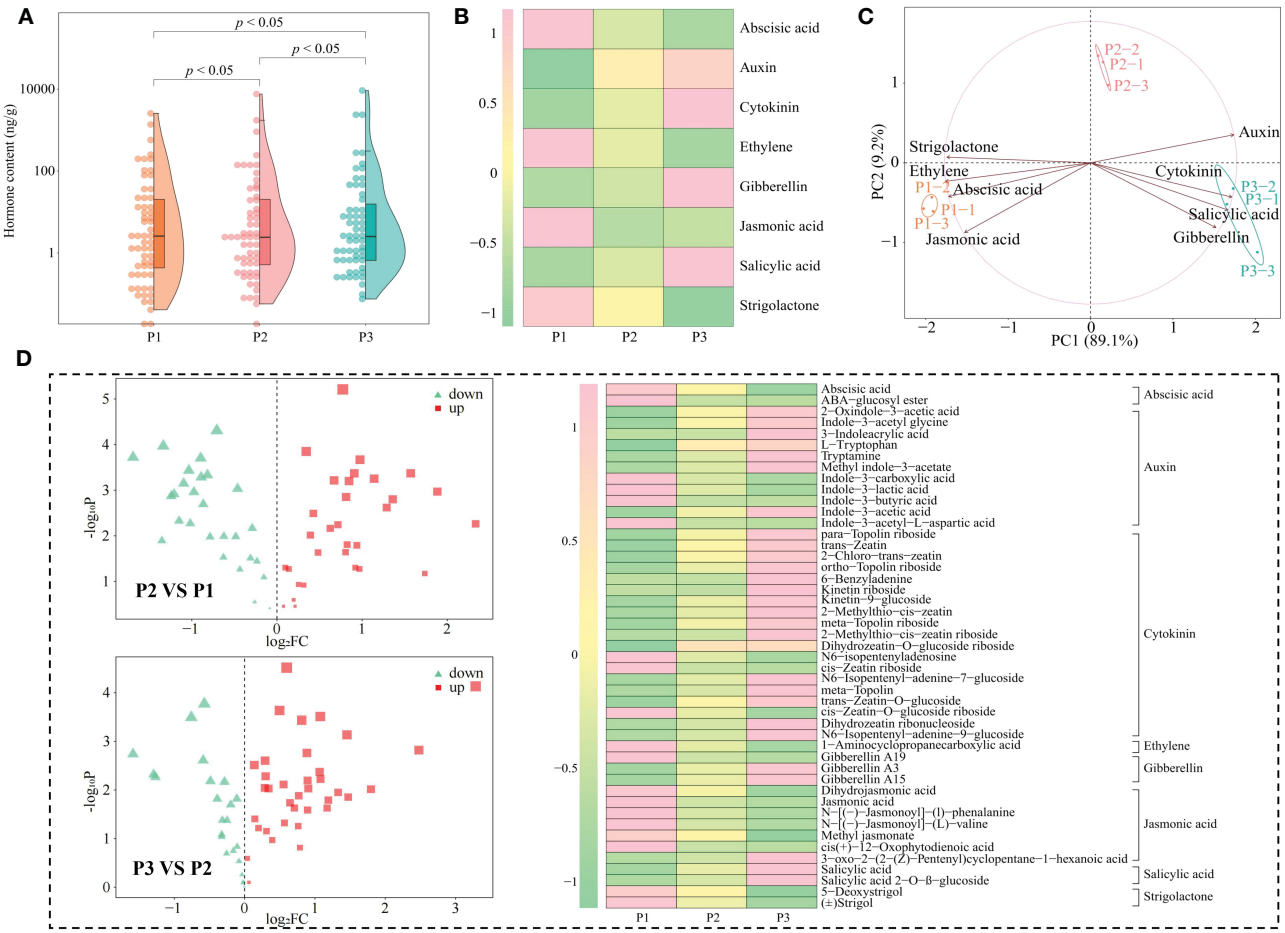
Hormone content analysis of tea tree leaves from soils with different pH. P1: Soil pH 3.29; P2: Soil pH 4.74; P3: Soil pH 5.32; **(A)** Effect of soil pH on the total hormone content of tea tree leaves; **(B)** Classification and content analysis of hormones in tea tree leaves; **(C)** PCA analysis of different categories of hormones in tea tree leaves; **(D)** Volcano and hot plot analysis of different hormone contents of tea tree leaves at different pH.

Phytohormones are key signaling compounds that regulate plant growth, development, and environmental stress responses (Wahab et al., 2022). In response to different types of abiotic stresses, plants need synthesize different types of hormones for self-regulation to adapt to their environment and thus safeguard their growth (Waadt et al., 2022). Acidification is also an abiotic stress on the tea tree itself, leading to reduced tea yield and quality (Ye et al., 2023a). In this study, we found that a total of 59 hormones were detected in tea tree leaves from soils with different pH values, and the hormone content showed a significant increase with the increase of soil pH, which was manifested by the total hormone content of 5.99 mg/g, 11.49 mg/g, and 15.41 mg/g when soil pH values were 3.29, 4.74, and 5.32, respectively (**Figure 4A**). Secondly, the results of the principal component analysis of the hormone content of tea tree leaves from soils with different pH showed (**Supplementary Figure 3**) that the two principal components could effectively differentiate the different samples, with 76.5% contribution from principal component 1 and 16.4% from principal component 2, giving a total contribution of 91.9%. Further analysis revealed that the 59 hormones detected could be categorized into eight groups, of which the content of auxin, cytokinin, gibberellin, and salicylic acid in tea tree leaves showed an increasing trend with the increase of soil pH, while the content of abscisic acid, ethylene, jasmonic acid, and strigolactone showed a decreasing trend (**Figure 4B**). PCA analysis showed (**Figure 4C**) that the content of auxin, cytokinin, gibberellin, and salicylic acid was significantly correlated with P1, whereas the content of abscisic acid, ethylene, jasmonic acid, and strigolactone was significantly correlated with P3. Further volcano plot analysis (**Figure 4D**) showed significant changes in the content of 47 hormones, with 19 hormones showing a decreasing trend and 28 hormones showing an increasing trend with the increase of soil pH. Further categorical and PCA analysis of the 47 hormones showed (**Supplementary Figure 4**) that they could be classified into eight categories and still showed that the contents of auxin, cytokinin, gibberellin, and salicylic acid were significantly correlated with P1, whereas the contents of abscisic acid, ethylene, jasmonic acid, and strigolactone were significantly correlated with P3.

### 3.5 Screening of characteristic hormones in tea tree leaves from soils with different pH

**Figure 5:**
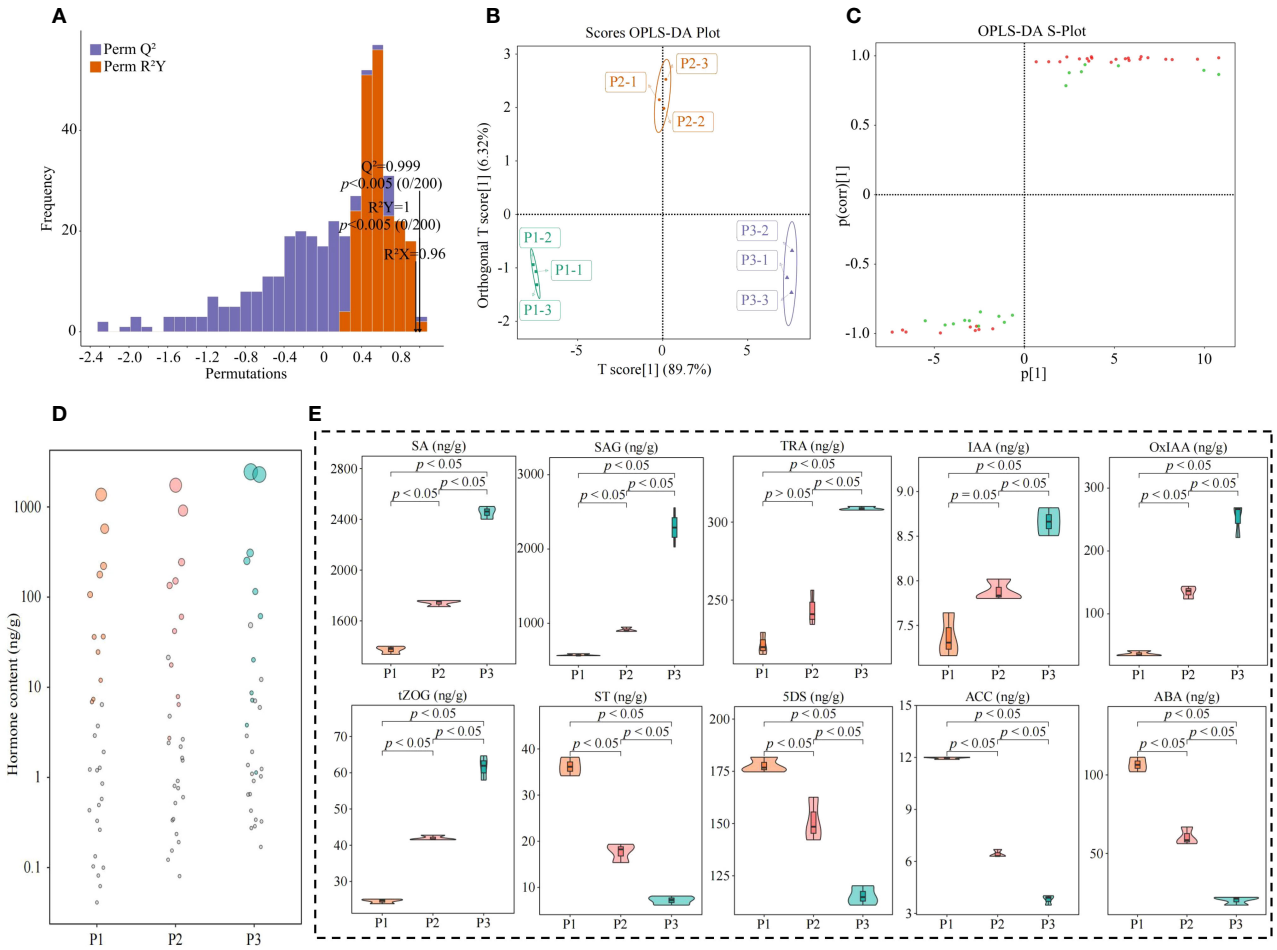
Characteristic hormone screening for significant changes in tea tree leaves in soils with different pH. P1: Soil pH 3.29; P2: Soil pH 4.74; P3: Soil pH 5.32; **(A)** OPLS-DA model test for screening of key hormones of tea tree leaves in soils with different pH; **(B)** Score plots of differences between groups of the OPLS-DA model for hormone content of tea tree leaves in soils with different pH; **(C)** S-Plot analysis of the OPLS-DA model for hormone content of tea tree leaves in soils with different pH; **(D)** Screening of characteristic hormones of tea tree leaves in soils with different pH based on bubble characteristic plot; **(E)** Content analysis of characteristic hormones.

The OPLS-DA model can be effectively used to screen for key indexes of major changes between samples. In this study, based on the hormone content in tea tree leaves, we further constructed the OPLS-DA model for soils with different pH, analyzed the model fit, differences between groups and screened for key hormones. The results of the OPLS-DA model fit analysis showed (**Figure 5A**) that after 200 random simulations, the goodness-of-fit R^2^Y value was 1, and the predictability Q^2^ value was 0.999, both of which reached highly significant levels (*p* < 0.005). It can be seen that the OPLS-DA model constructed in this study has a good fit and high confidence, which can effectively distinguish different samples and can be used for further analysis. The results of the score plot analysis of differences between groups of the OPLS-DA model showed (**Figure 5B**) that the OPLS-DA model could effectively distinguish samples, and the difference between groups of different samples reached 89.7%. It can be seen that there was a significant difference in the hormone content in the leaves of tea tree from soils with different pH. The results of the S-Plot analysis of the OPLS-DA model of the hormone content of tea tree leaves from soils with different pH values showed (**Figure 5C**) that a total of 30 key differential hormones were screened and obtained, of which, the contents of 21 hormones showed an increasing trend with the increase of soil pH, while the contents of the remaining 9 hormones showed a decreasing trend. Further bubble characteristic map of the 30 key hormones obtained a total of 10 significantly varying characteristic hormones (**Figure 5D**), of which 6 hormones (Tryptamine, 2-oxindole-3-acetic acid, indole-3-acetic acid, salicylic acid, salicylic acid 2-O-β-glucoside, and trans-zeatin-O-glucoside) showed a significant increasing trend with the increase of soil pH, while 4 hormones (5-Deoxystrigol, (±)strigol, abscisic acid, and 1-aminocyclopropanecarboxylic acid) showed a significant decreasing trend (**Figure 5E**).

Salicylic acid is a kind of phytohormone, which is mainly stored in the form of salicylic acid 2-O-β-glucoside in the plant, and increasing its content is conducive to the promotion of plant growth, and the improvement of the plant’s defense and disease resistance ability (Berim and Gang, 2022; Liu et al., 2023b). It can be seen that an increase in soil pH favors an increase in the synthesis of salicylic acid in the tea tree, which in turn induces an increase in the resistance of the tea tree, while the synthesized excess salicylic acid is stored in the leaves of the tea tree in the form of salicylic acid 2-O-β-glucoside. Tryptamine is a precursor for the synthesis of indole-3-acetic acid, which is oxidized to 2-oxindole-3-acetic acid, both of which can regulate plant growth and photosynthesis, and at the appropriate concentration can promote plant growth and improve the ability of plant photosynthesis (Bělonožníková et al., 2022; Tivendale and Millar, 2022). Trans-Zeatin-O-glucoside belongs to the phytokinin, which is beneficial to increase the proliferation of plant cells, and at the same time can increase the photosynthesis ability of plants (Abdouli et al., 2022). It can be seen that with the increase of soil pH, the content of tryptamine in tea tree leaves increased, providing a large amount of precursor substances for the synthesis of indole-3-acetic acid, and the oxidation of indole-3-acetic acid increased the content of 2-oxindole-3-acetic acid, which in turn promoted the growth of leaves and buds of the tea tree, and improved the yield of tea leaves. At the same time, increased content of trans-Zeatin-O-glucoside promoted the proliferation of tea tree cells and the growth of tea trees.

Secondly, it was also found in this study that the content of abscisic acid, 1-Aminocyclopropanecarboxylic acid, 5-deoxystrigol and (±)strigol in the leaves of tea tree was significantly reduced with increase in soil pH. Abscisic acid is commonly used to evaluate plant resistance to adversity stress, with higher levels indicating lower plant resistance to adversity (Muhammad Aslam et al., 2022). 1-Aminocyclopropanecarboxylic acid is a precursor to ethylene synthesis in plants, and its content increased significantly under adverse stresses (Gong et al., 2022). It can be seen that with the increase of soil pH, the content of abscisic acid and 1-Aminocyclopropanecarboxylic acid in the leaves of tea trees decreased, and the senescence of tea trees slowed down, which was conducive to improving the tea trees’ own resistance and guaranteeing the normal growth of tea trees. While, the main function of 5-deoxystrigol and (±)strigol is to stimulate the growth of parasitic plants in the periphery of the plant, and lowering their content is favorable to promote plant growth and increase plant yield (Hao et al., 2023; Zan et al., 2023a). Increasing soil pH reduced the content of 5-deoxystrigol and (±)strigol in tea tree leaves, which in turn affected the growth of the parasitic plants, reduced the competition for resources between the parasitic plants and the tea tree, and favored the promotion of tea tree growth.

### 3.6 Interaction analysis

**Figure 6:**
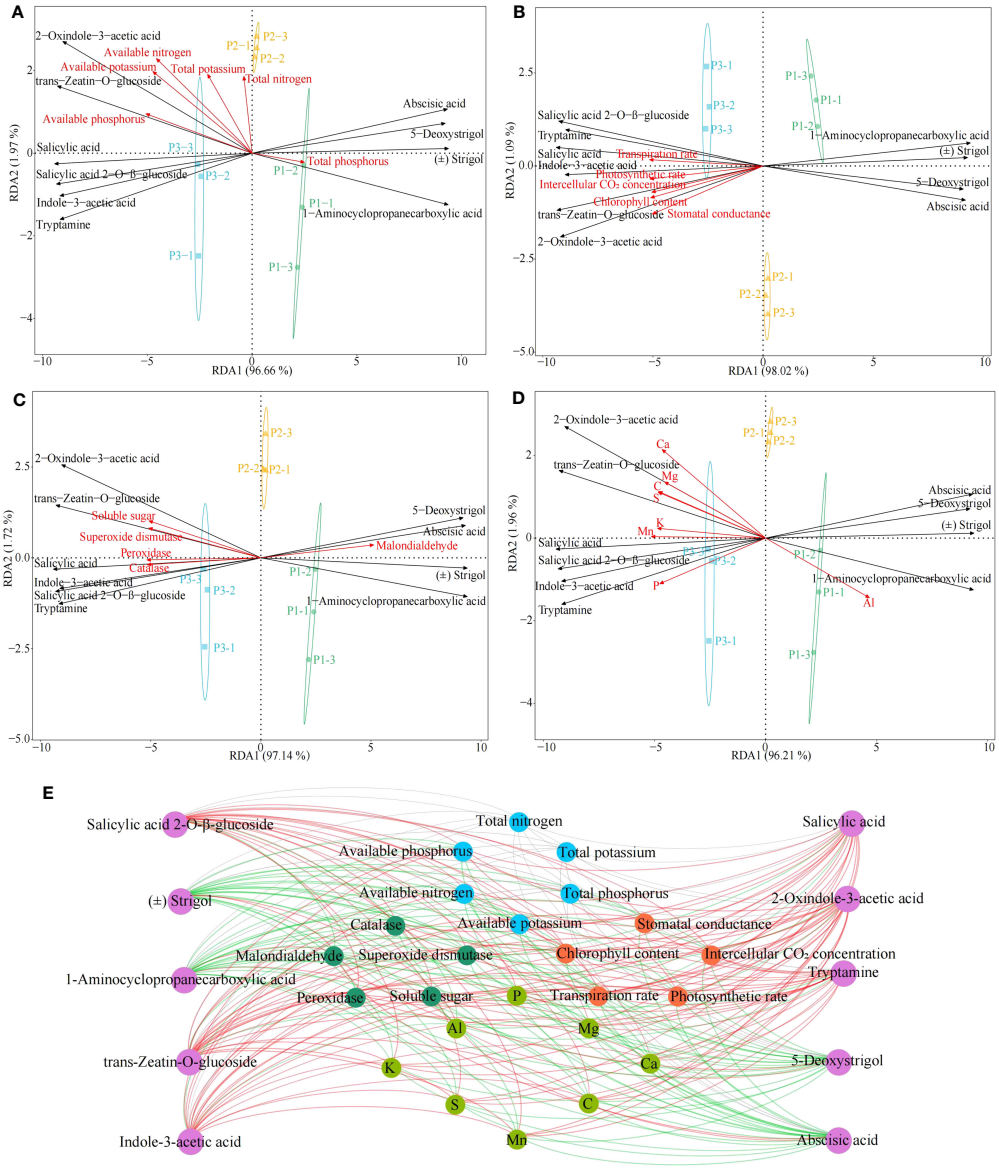
Interactions between tea tree leaves characteristic hormones, characteristic elements, physiological indexes and soil physicochemical index. P1: Soil pH 3.29; P2: Soil pH 4.74; P3: Soil pH 5.32; **(A)** Redundancy analysis of characteristic hormones and soil physicochemical indexes; **(B)** Redundancy analysis of characteristic hormones and photosynthetic physiological indexes; **(C)** Redundancy analysis of characteristic hormones and resistance physiological indexes; **(D)** Redundancy analysis of characteristic hormones and characteristic elements; **(E)** Interactions between different indexes. **―**Red lines represent significant positive correlations; **―**Green lines represent significant negative correlations; **―**Gray lines represent not significant correlations.

During growth, plants require a number of essential components from the external environment, including many elements, which are involved in the induction, synthesis and accumulation of phytohormones (Li et al., 2022). For example, Si activates phytohormone signaling mechanisms, Zn and Cu promote plant growth hormone production, and Cr promotes plant jasmonate synthesis (Awasthi et al., 2022; López-Bucio et al., 2022; Khan et al., 2023). Accordingly, on the basis of the previous study, this study further analyzed the interactions among tea tree leaves physiological indexes, characteristic elements, hormones and physicochemical indexes in soils with different pH. Redundancy analysis showed that six hormones in tea tree leaves, including salicylic acid, salicylic acid 2-O-β-glucoside, tryptamine, 2-oxindole-3-acetic acid, indole-3-acetic acid, trans-zeatin-O-glucoside, were significantly correlated with soil physicochemical indexes such as available nitrogen, available phosphorus, available potassium (**Figure 6A**), with photosynthetic physiological indexes such as photosynthetic rate, stomatal conductance, intercellular CO_2_ concentration, transpiration rate, chlorophyll content (**Figure 6B**), with resistance indexes such as superoxide dismutase, catalase, peroxidase, soluble sugar (**Figure 6C**), and with the content of Ca, Mg, C, S, K, Mn, and P (**Figure 6D**). Secondly, four hormones, 5-deoxystrigol, (±)strigol, abscisic acid, 1-aminocyclopropanecarboxylic acid, from tea tree leaves, were significantly correlated with malondialdehyde, total phosphorus and Al content.

Environmental changes have been reported to affect the uptake of mineral elements by plants and to alter the synthesis of different classes of hormones in plants, which in turn affects plant growth (Ulger et al., 2004). Keskin et al. (2022) found that indole-3-acetic acid, salicylic acid, cytokinin, and zeatin contents in plants were significantly and positively correlated with the contents of P, Mn, Ca, S, K, and Mg, as well as the activities of catalase, peroxidase, and superoxide dismutase, and significantly and negatively correlated with abscisic acid content. Liu et al. (2023a) found that under drought stress, plants inoculated with arbuscular mycorrhizal fungi effectively increased their uptake of P, K, Ca, Mg, and Mn, which in turn increased the content of brassinolide and indole-3-acetic acid in the plants, and promoted plant growth. Alp et al. (2023) found that at high concentrations of heavy metal stress, plant uptake of P, Mn, Ca, S, K, and Mg was reduced, and levels of hormones such as indole-3-acetic acid, cytokinin, and salicylic acid in plants were decreased, antioxidant enzyme activities in plants were reduced, photosynthesis capacity was decreased, and plant growth was impeded. This study found (**Figure 6E**, **Supplementary Figure 5**) that six hormones in tea tree leaves, including salicylic acid, salicylic acid 2-O-β-glucoside, tryptamine, 2-oxindole-3-acetic acid, indole-3-acetic acid, trans-zeatin-O-glucoside, were significantly correlated with soil available nutrient content such as available nitrogen, available phosphorus, available potassium, with photosynthetic physiological indexes such as photosynthetic rate, stomatal conductance, intercellular CO_2_ concentration, transpiration rate, chlorophyll content, with resistance indexes such as superoxide dismutase, catalase, peroxidase, soluble sugar, and with the content of Ca, Mg, C, S, K, Mn, and P; while four hormones, 5-deoxystrigol, (±)strigol, abscisic acid, 1-aminocyclopropanecarboxylic acid, from tea tree leaves were significantly correlated with malondialdehyde, a physiological index of resistance, and significantly correlated with Al content. It can be seen that the increase of soil pH was conducive to improving the rhizosphere soil available nutrient content of the tea tree, promoting the growth of the tea tree root system, and then improving the tea tree’s absorption and accumulation of Ca, Mg, C, S, K, Mn, P in the soil, and enhancing the synthesis and accumulation of hormones in the tea tree leaves such as salicylic acid, salicylic acid 2-O-β-glucoside, tryptamine, 2-oxindole-3-acetic acid, indole-3-acetic acid, trans-zeatin-O-glucoside, which in turn improved the tea tree’s own resistance and photosynthesis ability and promoted the growth of the tea tree.

## 4 Conclusion

**Figure 7:**
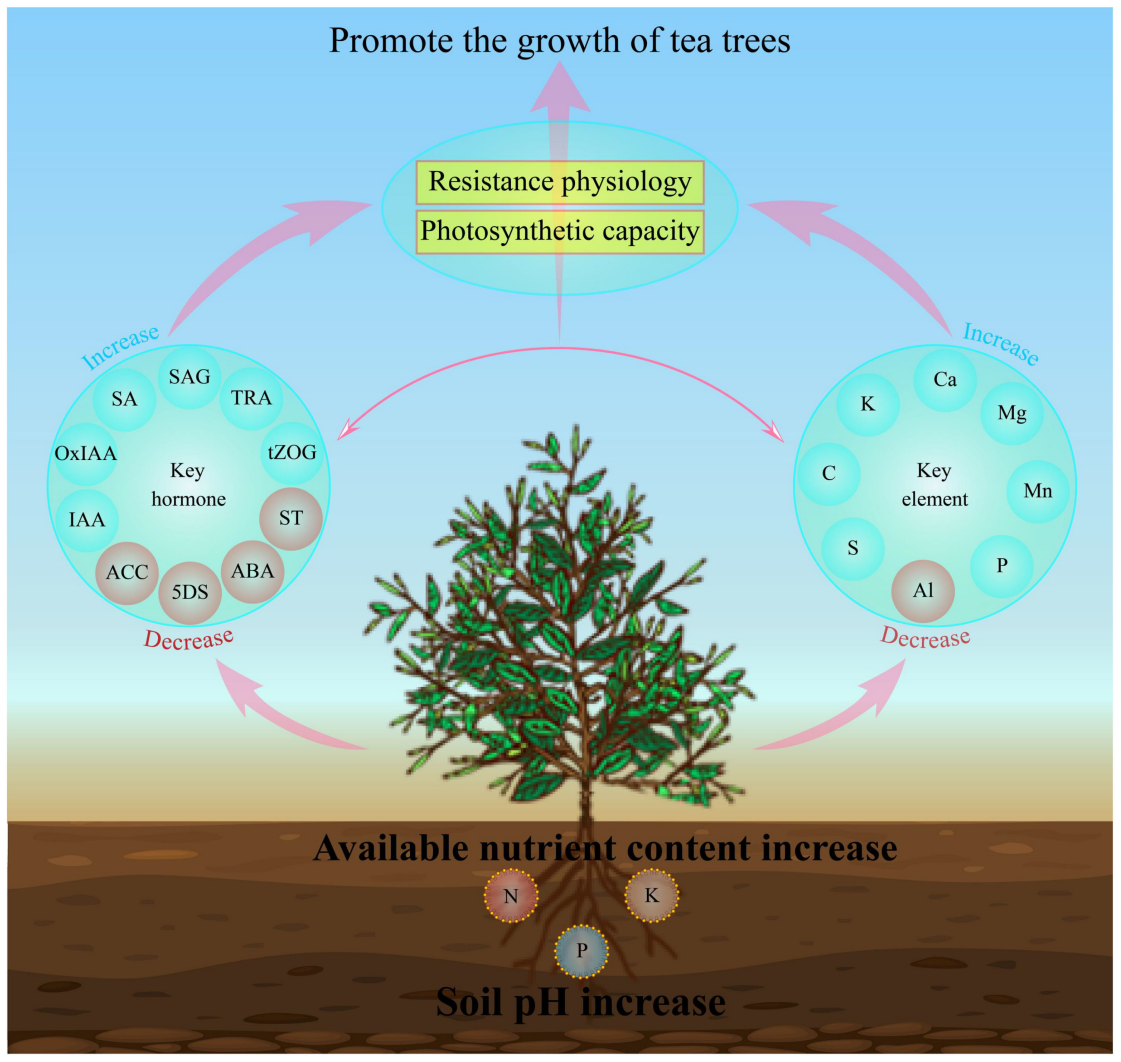
Mechanisms of the effect of soil pH on elemental uptake, hormone synthesis and physiological characteristics of tea tree leaves. IAA, Indole-3-acetic acid; OxIAA, 2-oxindole-3-acetic acid; SA, Salicylic acid; SAG, Salicylic acid 2-O-β-glucoside; TRA, Tryptamine; tZOG, trans-Zeatin-O-glucoside; ACC, 1-Aminocyclopropanecarboxylic acid; 5DS: 5-Deoxystrigol; ABA, Abscisic acid; ST, (±)Strigol.

In this study, we analyzed the effects of soils with different pH on soil physicochemical indexes, physiological indexes, multi-element content, hormone metabolome of tea tree leaves and their interactions. The results showed (**Figure 7**) that with the increase of soil pH, the available nutrient content of the rhizosphere soil of the tea tree rose, which was conducive to promoting root growth of the tea tree. At the same time, it promoted the uptake and accumulation of C, K, Ca, Mg, Mn, P and S in the tea tree, and reduced the enrichment of Al. It enhanced the synthesis and accumulation of hormones in the leaves of tea trees such as salicylic acid, salicylic acid 2-O-β-glucoside, tryptamine, 2-oxindole-3-acetic acid, indole-3-acetic acid, trans-zeatin-O-glucoside, and reduced the content of 5-deoxystrigol, (±)strigol, abscisic acid, 1-aminocyclopropanecarboxylic acid, which in turn enhanced the resistance of the tea tree to the environment, increased the antioxidant enzyme activity and photosynthesis capacity of the tea tree, and promoted the growth of the tea tree. This study analyzed the effects of soil pH on tea tree growth from the perspectives of physiological properties, elemental uptake and hormone metabolomes, which is of great significance for soil remediation of acidified tea plantations and exogenous regulation of tea tree growth.

## Data availability statement

The datasets presented in this study can be found in online repositories. The names of the repository/repositories and accession number(s) can be found in the article/**Supplementary Material**.

## Author contributions

XJ: Conceptualization, Formal Analysis, Funding acquisition, Methodology, Visualization, Writing – original draft, Writing – review & editing. QZ: Conceptualization, Formal Analysis, Funding acquisition, Methodology, Visualization, Writing – original draft, Writing – review & editing. YW: Formal Analysis, Writing – review & editing. YZ: Formal Analysis, Writing – review & editing. ML: Formal Analysis, Writing – review & editing. PC: Investigation, Methodology, Writing – original draft. MC: Investigation, Methodology, Writing – original draft. SL: Investigation, Methodology, Writing – original draft. JZ: Investigation, Methodology, Writing – original draft. JY: Conceptualization, Formal Analysis, Methodology, Project administration, Resources, Supervision, Visualization, Writing – original draft, Writing – review & editing. HW: Conceptualization, Formal Analysis, Methodology, Project administration, Resources, Supervision, Visualization, Writing – original draft, Writing – review & editing.
