# Supplementary material for: Changes of physiological characteristics, element accumulation and hormone metabolism of tea leaves in response to soil pH
Source: Front Plant Sci. 2023 Nov 16;14:1266026. doi: 10.3389/fpls.2023.1266026 (PMC10687463; doi:10.3389/fpls.2023.1266026)
Supplement: Supplementary file 1 [file DataSheet_1.pdf]

Supplementary Materials

Table S1 Comparison of values of tea standard (GBW10016) determined by ICP-MS with certified values

Element	Certified value	Measured value	Recovery (%)	Element	Certified value	Measured value	Recovery (%)
Li	0.25±0.02	0.242±0.012	96.80±4.80	Nd	1.0±0.1	0.944±0.045	94.40±4.50
Be	50±8	48.323±1.243	96.65±2.49	Sm	0.12±0.02	0.116±0.004	96.67±3.33
B	12.6±0.8	11.842±0.625	93.98±4.96	Eu	27±2	24.375±0.893	90.28±3.31
Na	22.6±6.7	21.465±0.871	94.98±3.85	Gd	0.14±0.02	0.122±0.003	87.14±2.14
Mg	1.86±0.11	1.736±1.222	93.33±6.56	Tb	21±2	18.927±0.985	90.13±4.69
Si	0.42±0.12	0.393±0.023	93.57±5.48	Dy	0.13±0.02	0.141±0.003	108.46±2.31
N	34.2±3.5	36.184±0.952	105.80±2.78	Ho	28±4	25.382±1.025	90.65±3.66
P	2.42±0.13	2.021±0.051	83.51±2.11	Er	0.08±0.01	0.083±0.004	103.75±103.75
K	14.53±0.52	13.897±0.652	95.64±4.49	Tm	14±2	13.015±0.429	92.96±3.06
Sc	89±17	82.675±3.165	92.89±3.56	Yb	0.10±0.02	0.092±0.003	92.00±3.00
Ti	11	9.954±0.735	90.49±6.68	Lu	16±3	14.254±0.784	89.09±4.90
V	0.3±0.1	0.278±0.022	92.67±7.33	Hf	0.14±0.02	0.152±0.008	108.57±5.71
Cr	0.79±0.11	0.713±0.042	90.25±5.32	W	0.06	0.063±0.004	105.00±6.67
Mn	1.25±0.42	1.182±0.063	94.56±5.34	Hg	8±1	7.347±0.283	91.84±3.54
Fe	149±7	137.245±5.232	92.11±3.51	Tl	47±8	43.285±1.267	92.10±2.71
Co	0.28±0.02	0.253±0.021	90.36±7.50	Pb	1.09±0.13	1.052±0.049	96.51±4.50
Ni	3.4±0.3	3.434±0.118	101.00±3.47	Bi	0.023±0.005	0.021±0.002	91.30±8.70
Cu	8.3±0.5	8.413±0.265	101.36±3.19	Th	33.4±7.2	30.871±1.032	92.43±3.09
Zn	27±3	25.826±0.617	95.65±2.29	U	13±2	12.019±0.543	92.45±4.18
Ge	13±5	11.195±0.353	86.12±2.72	Al	2.54±0.22	2.235±0.134	87.99±5.28
As	0.10±0.02	0.084±0.005	84.00±5.00	S	2.62±0.21	2.383±0.109	90.95±4.16
Br	3	2.756±0.086	91.87±2.87	Ca	4.73±0.25	4.092±0.193	86.51±4.08
Rb	45.9±3.3	41.653±1.173	90.75±2.56	Sb	0.05±0.01	0.046±0.003	92.00±6.00
Sr	9.1±12	8.264±0.382	90.81±4.20	Rh	—	—	—
Y	2.0±0.2	1.846±0.065	92.30±3.25	Pd	—	—	—
Nb	0.66±0.08	0.571±0.031	86.52±4.70	In	—	—	—
Mo	0.04±0.01	0.041±0.001	102.50±2.50	Te	—	—	—
Ag	0.010±0.002	0.009±0.000	90.00±3.00	C	—	—	—
Cd	46±5	43.143±1.421	93.79±3.09	Ga	—	—	—
Sn	0.06±0.01	0.062±0.002	103.33±3.33	Zr	—	—	—
I	(0.2)	0.182±0.006	91.00±3.00	Ta	—	—	—
Cs	0.19±0.02	0.173±0.013	91.05±6.84	Os	—	—	—
Ba	32±3	30.152±0.862	94.23±2.69	Ir	—	—	—
La	1.01±0.08	1.036±0.032	102.57±3.17	Pt	—	—	—
Ce	1.3±0.2	1.245±0.057	95.77±4.38	Ru	—	—	—
Pr	0.16±0.02	0.171±0.003	106.88±1.88				

Note: Means ± standard error (SE) from three replications for each sample is shown. For GBW10016, the units of Mg, Si, N, P, K, Mn, Al, S, and Ca are mg/g; the units of Be, Sc, Cd, Eu, Tb, Ho, Lu, Hg, Tl, Bi, Th, and U are μg/kg; and those of other elements are μg/g, respectively.

Table S2 Comparison of standard values and certified values of eight soil elements determined by ICP-MS

Element	Certified value (mg/g)	Measured value (mg/g)	Recovery (%)
C	0.035±0.001	0.036±0.002	102.86±5.71
Mg	0.348±0.03	0.321±0.009	92.24±2.59
P	0.32±0.008	0.335±0.009	104.78±3.01
K	1.261±0.002	1.198±0.034	95.00±2.70
Mn	0.304±0.021	0.296±0.007	97.52±2.25
Al	6.48±0.07	6.070±0.236	93.67±3.64
S	0.12±0.02	0.117±0.005	97.82±4.41
Ca	0.907±0.043	0.892±0.057	98.35±6.28

Note: Means ± standard error (SE) from three replications for each sample is shown.

Table S3 Standard curves for different hormones

Index	Class	RT	Equation	r	LLOQ	ULOQ
ABA-glucosyl ester	ABA	4.47	y = 0.00151 x + 9.32183e-5	0.99151	5	500
Abscisic acid	ABA	5.26	y = 0.11670 x + 5.34143e-4	0.99754	0.1	500
L-tryptophan	Auxin	3.33	y = 3838.12685 x + 7789.66169	0.99942	1	10000
Tryptamine	Auxin	3.63	y = 6.55464e4 x + 11685.45074	0.99432	0.1	500
2-oxindole-3-acetic acid	Auxin	4.21	y = 0.00924 x + 2.82467e-4	0.99987	1	500
Indole-3-acetyl-L-aspartic acid	Auxin	4.27	y = 0.02116 x + 3.35390e-4	0.99713	0.1	500
1-O-indol-3-ylacetylglucose	Auxin	4.28	y = 3.67456e-5 x + 1.48106e-5	0.99474	1	500
3-Indoleacetamide	Auxin	4.31	y = 0.02550 x + 7.39621e-4	0.99173	0.1	500
Indoleacetyl glutamic acid	Auxin	4.39	y = 0.03746 x + 1.14163e-4	0.99758	0.1	500
Indole-3-acetyl glycine	Auxin	4.40	y = 0.06853 x + 0.00349	0.99725	1	500
Indole-3-lactic acid	Auxin	4.62	y = 0.00449 x + 0.00130	0.99864	2	500
N-(3-Indolylacetyl)-L-alanine	Auxin	4.70	y = 0.10307 x + 0.00138	0.99899	0.1	500
Indole-3-carboxylic Acid	Auxin	4.75	y = 0.01025 x + 2.88715e-4	0.99946	0.5	500
Indole-3-carboxaldehyde	Auxin	4.88	y = 0.02292 x + 0.00172	0.99965	0.1	500
Indole-3-acetic acid	Auxin	5.00	y = 0.01244 x + 2.18869e-4	0.99597	0.2	500
3-Indoleacrylic acid	Auxin	5.22	y = 0.01272 x + 2.40081e-4	0.99950	0.2	500
N-(3-Indolylacetyl)-L-valine	Auxin	5.36	y = 0.17292 x + 0.00115	0.99613	0.1	500
3-Indolepropionic acid	Auxin	5.41	y = 0.03641 x - 7.07410e-4	0.99855	0.1	500
Indole-3-acetyl-Lglutamic acid dimethyl ester	Auxin	5.53	y = 0.01351 x + 1.38597e-4	0.99776	0.5	500
Indole-3-acetyl-L-tryptophan	Auxin	5.70	y = 0.06755 x + 5.56844e-4	0.99995	0.1	500
N-(3-Indolylacetyl)-L-leucine	Auxin	5.72	y = 0.24042 x + 0.00625	0.99379	0.1	500
3-Indolebutyric acid	Auxin	5.72	y = 0.02094 x + 0.02575	0.99902	0.2	500
3-Indoleacetonitrile	Auxin	5.75	y = 0.00920 x + 7.48926e-4	0.99987	0.5	500
N-(3-Indolylacetyl)-L-phenylalanine	Auxin	5.81	y = 0.11778 x + 0.00173	0.99993	0.5	500

Methyl indole-3-acetate	Auxin	6.04	y = 0.04956 x + 3.23136e-4	0.99067	0.2	500
Indole-3-acetyl-L-valine methyl ester	Auxin	6.07	y = 0.77422 x + 0.00849	0.99127	0.1	500
Indole-3-acetyl-L-leucine methyl ester	Auxin	6.41	y = 0.72903 x + 0.00597	0.99007	0.1	500
Indole-3-acetyl-L-phenylalanne methyle ester	Auxin	6.45	y = 0.63263 x + 0.00509	0.99285	0.1	500
trans-Zeatin-O-glucoside	Cytokinins	2.79	y = 0.04821 x + 7.84255e-4	0.99918	0.2	500
trans-Zeatin	Cytokinins	3.02	y = 0.16189 x + 0.00925	0.99969	0.1	500
Dihydrozeatin	Cytokinins	3.16	y = 0.07216 x + 0.00333	0.99948	0.1	500
Dihydrozeatin-7-glucoside	Cytokinins	3.20	y = 0.39834 x + 0.00183	0.99754	0.1	500
cis-Zeatin	Cytokinins	3.25	y = 0.08311 x + 0.00494	0.99878	0.1	500
cis-Zeatin-9-glucoside	Cytokinins	3.30	y = 0.39242 x + 4.21780e-4	0.99275	0.1	500
Dihydrozeatin-O-glucoside riboside	Cytokinins	3.51	y = 0.21989 x + 6.96833e-4	0.99588	0.1	500
cis-Zeatin-O-glucoside riboside	Cytokinins	3.54	y = 0.05670 x + 4.76908e-5	0.99878	0.1	500
4-[[(9-beta-D-Glucopyranosyl-9H-purin-6-yl)amino]methyl]phenol	Cytokinins	3.58	y = 0.17326 x + 3.87279e-4	0.99366	0.1	500
para-Topolin	Cytokinins	3.58	y = 0.07163 x + 9.45010e-4	0.99880	0.2	500
trans-Zeatin riboside	Cytokinins	3.61	y = 0.33210 x + 0.00101	0.99610	0.1	500
Dihydrozeatin ribonucleoside	Cytokinins	3.62	y = 0.18714 x + 7.25188e-4	0.99237	0.1	500
cis-Zeatin riboside	Cytokinins	3.70	y = 0.19371 x + 0.00131	0.99518	0.1	500
N6-Isopentenyl-adenine-7-glucoside	Cytokinins	3.73	y = 0.41890 x + 0.00138	0.99984	0.1	500
meta-Topolin-9-glucoside	Cytokinins	3.74	y = 0.18541 x + 0.00116	0.99405	0.1	500
meta-Topolin	Cytokinins	3.74	y = 0.10091 x + 0.00137	0.99923	2	500
Kinetin-9-glucoside	Cytokinins	3.81	y = 0.25268 x + 0.00141	0.99330	0.1	500
Kinetin	Cytokinins	3.82	y = 0.07683 x + 9.12811e-4	0.99955	0.1	500
N6-Benzyladenine-7-glucoside	Cytokinins	3.83	y = 0.56164 x + 0.00418	0.99975	0.1	500
para-Topolin riboside	Cytokinins	3.95	y = 0.19060 x + 0.00142	0.99933	0.1	500
N6-Isopentenyl-adenine-9-glucoside	Cytokinins	4.09	y = 0.28146 x + 4.79044e-4	0.99061	0.1	500
ortho-Topolin-9-glucoside	Cytokinins	4.10	y = 0.14433 x + 3.80111e-4	0.99594	0.1	500

meta-Topolin riboside	Cytokinins	4.10	y = 0.32897 x + 7.59431e-4	0.99012	0.1	500
N6-isopentenyladenine	Cytokinins	4.14	y = 0.09525 x + 0.00121	0.99174	0.1	500
ortho-Topolin	Cytokinins	4.15	y = 0.17088 x + 6.50455e-4	0.99259	0.1	500
Kinetin riboside	Cytokinins	4.23	y = 0.31455 x + 3.64184e-4	0.99056	0.1	500
N6-Benzyladenine -9-glucoside	Cytokinins	4.25	y = 0.40849 x + 0.00252	0.99059	0.1	500
6-Benzyladenine	Cytokinins	4.31	y = 0.17423 x + 0.00223	0.99111	0.1	500
2-Chloro-trans-zeatin	Cytokinins	4.43	y = 0.07569 x + 5.69721e-4	0.99225	0.1	500
ortho-Topolin riboside	Cytokinins	4.47	y = 0.26717 x + 0.00247	0.99845	0.1	500
N6-isopentenyladenosine	Cytokinins	4.51	y = 0.10840 x + 8.51558e-4	0.99156	0.1	500
2-methylthio-cis-zeatin	Cytokinins	4.52	y = 0.04692 x + 3.64952e-4	0.99870	0.1	500
2-Methylthio-cis-zeatin riboside	Cytokinins	4.57	y = 0.19138 x + 0.00240	0.99650	0.1	500
6-Benzyladenosine	Cytokinins	4.65	y = 0.58678 x + 0.00418	0.99029	0.1	500
2-Methylthio-N6-isopentenyladenosine	Cytokinins	5.73	y = 0.04382 x + 4.85798e-4	0.99939	0.1	500
2-Methylthio-N6-isopentenyladenine	Cytokinins	5.84	y = 0.03323 x + 0.00107	0.99837	0.1	500
1-Aminocyclopropanecarboxylic acid	Ethylene	0.74	y = 13977.79709 x + 14178.28663	0.99931	1	500
Gibberellin A3	Gibberellins	4.43	y = 0.05119 x + 0.02846	0.99753	1	500
Gibberellin A1	Gibberellins	4.47	y = 0.01762 x + 0.01275	0.99658	2	500
Gibberellin A19	Gibberellins	5.13	y = 0.04180 x + 0.00196	0.99402	5	500
Gibberellin A20	Gibberellins	5.32	y = 0.08253 x + 0.00392	0.99807	2	500
Gibberellin A53	Gibberellins	5.70	y = 0.07635 x + 0.04869	0.99610	2	500
Gibberellin A7	Gibberellins	6.04	y = 0.35547 x + 0.00473	0.99360	1	500
Gibberellin A4	Gibberellins	6.12	y = 0.04612 x + 0.00219	0.99843	2	500
Gibberellin A24	Gibberellins	6.30	y = 0.08504 x + 8.10155e-4	0.99073	2	500
Gibberellin A15	Gibberellins	6.84	y = 0.06608 x + 0.00145	0.99986	2	500
Gibberellin A9	Gibberellins	6.84	y = 0.10601 x + 0.00296	0.99809	2	500
Jasmonic Acid	Jasmonates	5.76	y = 0.08013 x + 0.00434	0.99908	0.2	500

N-[(-)-Jasmonoyl]-(L)-valine	Jasmonates	5.96	y = 0.96511 x + 0.00382	0.99167	0.1	500
Dihydrojasmonic acid	Jasmonates	6.12	y = 0.11283 x + 0.00364	0.99422	0.5	500
Jasmonoyl-L-isoleucine	Jasmonates	6.29	y = 0.28397 x + 0.00186	0.99139	0.1	500
N-[(-)-Jasmonoyl]-(l)-phenalanine	Jasmonates	6.34	y = 0.71968 x + 0.00763	0.99008	0.1	500
3-oxo-2-(2-(Z)-Pentenyl) cyclopentane-1-butyric acid	Jasmonates	6.36	y = 0.06731 x + 0.04843	0.99361	2	500
Methyl jasmonate	Jasmonates	6.88	y = 0.60087 x + 0.01723	0.99959	0.2	500
3-oxo-2-(2-(Z)-Pentenyl)cyclopentane-1-hexanoic acid	Jasmonates	6.99	y = 0.04056 x + 0.01111	0.99760	5	500
cis(+)-12-Oxophytodienoic acid	Jasmonates	7.23	y = 1.88724 x + 0.05384	0.99026	0.1	500
Salicylic acid 2-O-β-Glucoside	Salicylic acid	3.57	y = 0.03450 x + 0.00254	0.99503	1	10000
Salicylic Acid	Salicylic acid	5.03	y = 0.10099 x + 0.01138	0.99994	0.5	500
（±）Strigol	Strigolactones	6.34	y = 589.53527 x + 178.11343	0.99650	10	500
5-Deoxystrigol	Strigolactones	7.46	y = 18617.98977 x + 2790.18589	0.99740	2	500

Note: Index: Hormone name; Class: Classification of hormones; RT: Retention time; Equation: Linear equation; r: Coefficient of Correlation; LLOQ: Lower limit of quantitation (ng/mL); ULOQ: Higher limit of quantitation (ng/mL).

Table S4 Analysis of eight elements in rhizosphere soil of tea tree at different pH values (mg/kg)

Element	P1	P2	P3
C	135.842±12.365 a	143.257±8.963 a	140.794±7.256 a
Mg	89.267±7.238 a	94.365±5.384 a	92.348±8.263 a
P	131.287±2.619 a	130.265±4.520 a	128.539±3.582 a
K	7410.268±154.987 a	7568.327±189.357 a	7492.538±163.245 a
Mn	26.184±2.136 a	25.149±1.986 a	24.185±3.485 a
Al	359.258±11.265 a	368.186±15.382 a	358.036±14.297 a
S	286.469±8.358 a	300.432±11.356 a	297.262±5.823 a
Ca	2456.275±123.654 a	2561.476±169.784 a	2523.465±103.268 a

Note: P1: Soil pH 3.29; P2: Soil pH 4.74; P3: Soil pH 5.32; Means ± standard error (SE) from three replications for each sample is shown; The same lowercase letter indicates that the difference between different samples does not reach the $p < 0.05$ level.

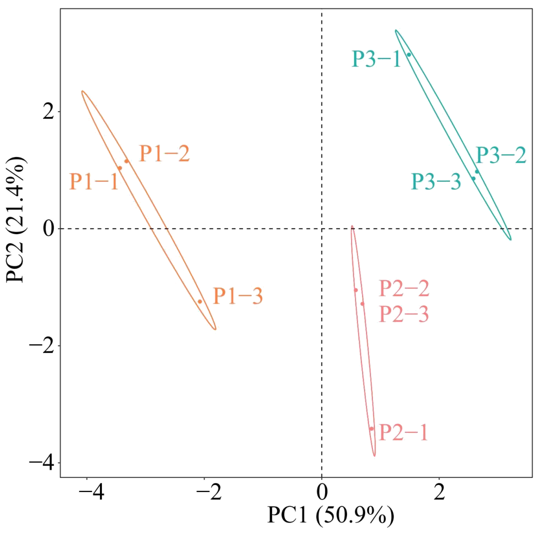

Fig. S1 PCA analysis of the effect of soil pH on the elemental content of tea tree leaves

Note: P1: Soil pH 3.29; P2: Soil pH 4.74; P3: Soil pH 5.32.

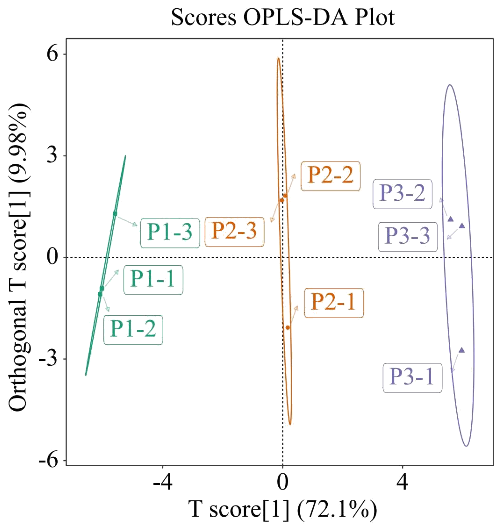

Fig. S2 Score plot analysis of differences between groups of element contents of tea samples from soils with different pH based on the OPLS-DA model

Note: P1: Soil pH 3.29; P2: Soil pH 4.74; P3: Soil pH 5.32.

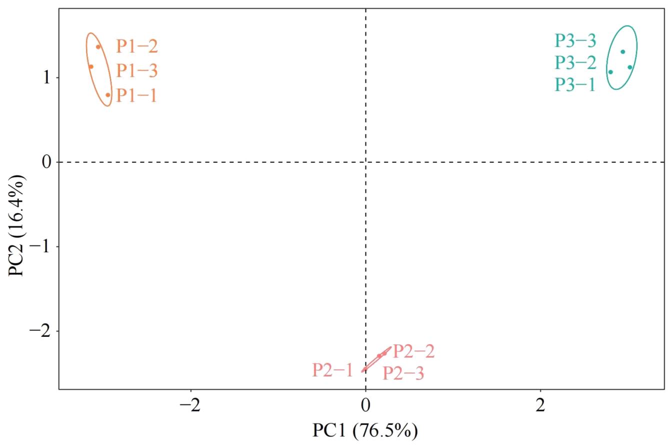

Fig. S3 PCA analysis of the effect of soil pH on the hormone content of tea tree leaves

Note: P1: Soil pH 3.29; P2: Soil pH 4.74; P3: Soil pH 5.32.

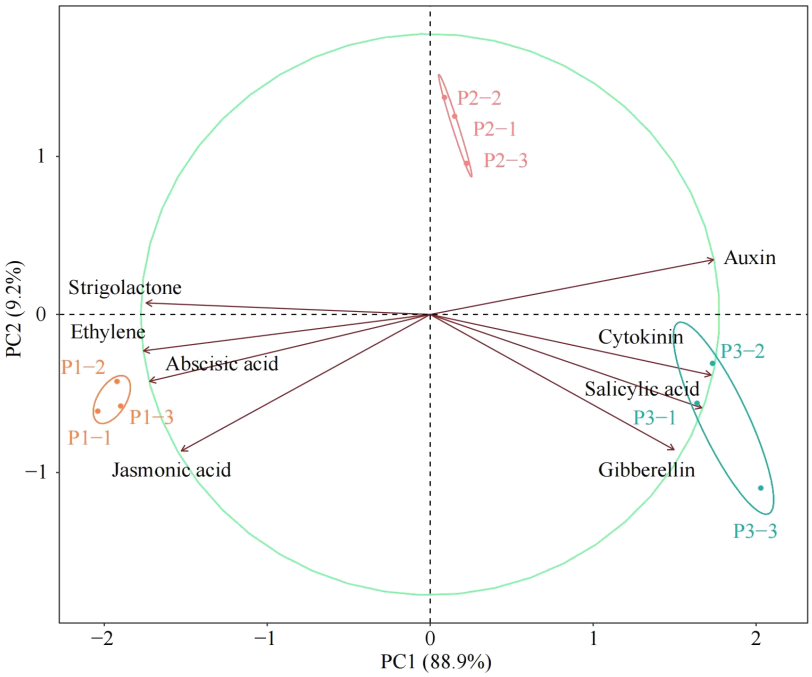

Fig. S4 Classification and PCA analysis of differential hormones in tea tree leaves at different pH

Note: P1: Soil pH 3.29; P2: Soil pH 4.74; P3: Soil pH 5.32.

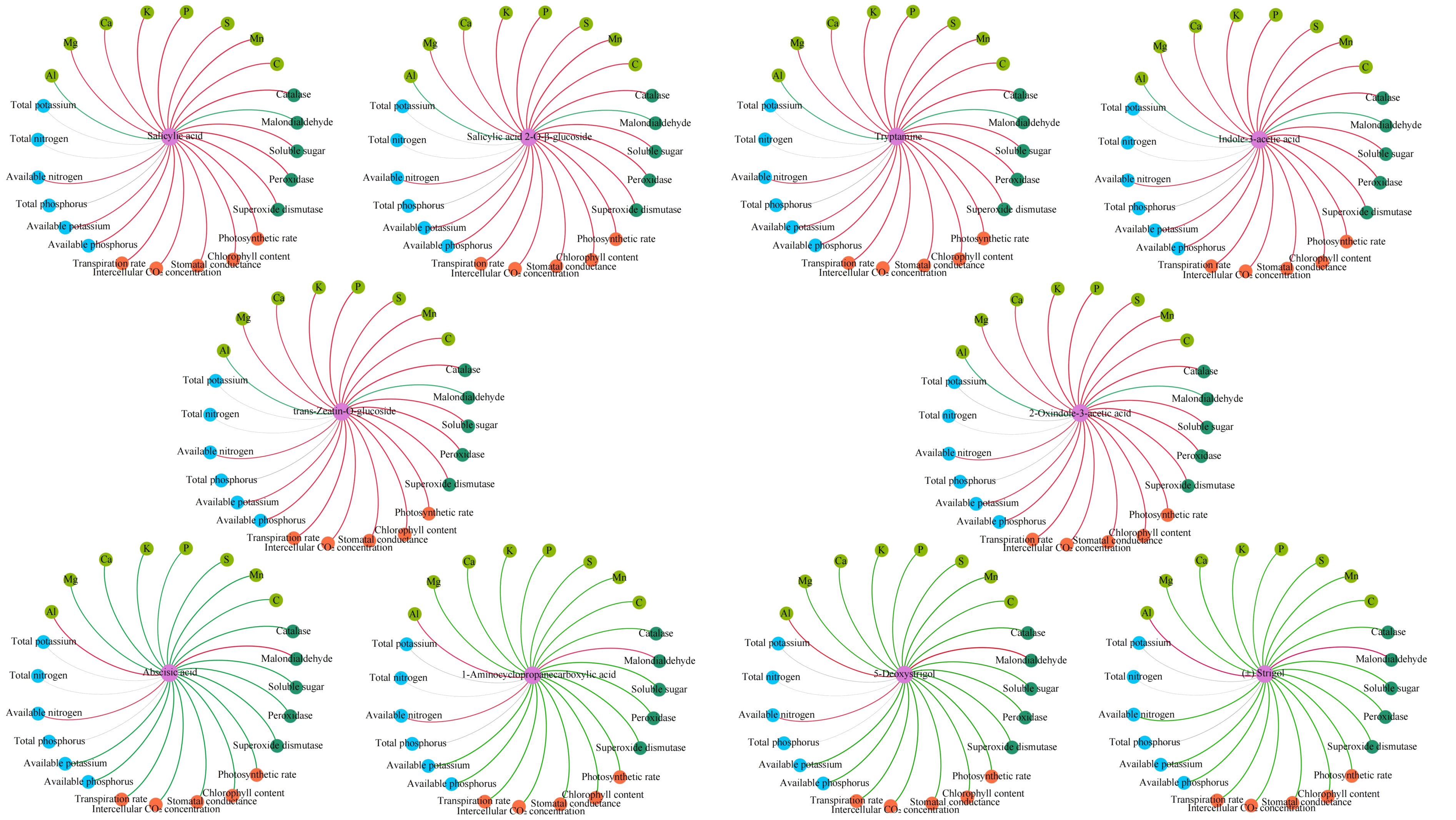

Fig. S5 Interactions between characteristic hormones, soil physicochemical indexes, characteristic elements and physiological indexes
